# Predicting evolution over multiple generations in deteriorating environments using evolutionarily explicit Integral Projection Models

**DOI:** 10.1111/eva.13272

**Published:** 2021-07-27

**Authors:** Tim Coulson, Tomos Potter, Anja Felmy

**Affiliations:** ^1^ Department of Zoology University of Oxford Oxford UK

**Keywords:** additive genetic variance, covariance, environmental change, Integral Projection Models, selection

## Abstract

Human impacts on the natural world often generate environmental trends that can have detrimental effects on distributions of phenotypic traits. We do not have a good understanding of how deteriorating environments might impact evolutionary trajectories across multiple generations, even though effects of environmental trends are often significant in the statistical quantitative genetic analyses of phenotypic trait data that are used to estimate additive genetic (co)variances. These environmental trends capture reaction norms, where the same (average) genotype expresses different phenotypic trait values in different environments. Not incorporated into the predictive models typically parameterised from statistical analyses to predict evolution, such as the breeder's equation. We describe how these environmental effects can be incorporated into multi‐generational, evolutionarily explicit, structured population models before exploring how these effects can influence evolutionary dynamics. The paper is primarily a description of the modelling approach, but we also show how incorporation into models of the types of environmental trends that human activity has generated can have considerable impacts on the evolutionary dynamics that are predicted.

## 1 INTRODUCTION

To predict how populations will be impacted by human‐induced environmental change, it is necessary to understand how their numerical dynamics will be altered (Chevin et al., 2010; Coulson et al., 2011). One way to do this is to ask how human‐induced biotic and abiotic environmental change will affect the survival and reproductive rates that determine temporal variation in population growth and fitness (Tuljapurkar, 2013; Tuljapurkar & Caswell, 2012). These rates are functions of (i) ecosystem, community and population level processes and (ii) individual attributes such as age, sex and phenotypic trait values (Ellner et al., 2016). The phenotypic traits that contribute to determining survival and reproductive rates are, by definition, fitness‐related traits under selection (Lande, 1982). The functions that link phenotypic trait values to survival and recruitment are termed fitness functions. Any human‐induced biotic or abiotic environmental driver that alters survival and recruitment consequently has the potential to alter selection pressures and the rate, and potentially direction, of evolution. Evolution of these fitness‐related traits in response to human‐induced environmental change is a type of biotic change and can in turn influence survival and recruitment rates, and consequently the population dynamics, generating eco‐evolutionary feedbacks (Hendry, 2016).

Human‐induced biotic and abiotic environmental change can also impact phenotypic traits via phenotypic plasticity and nongenetic inheritance (Reed et al., 2011; Salinas et al., 2013; Via & Lande, 1985). These processes alter the map between genotype and phenotypic trait value such that the same genotype may generate different phenotypic trait values in different environments. The difference between the two is that phenotypic plasticity leads to environment‐induced phenotypic changes in the individual experiencing environmental change, while nongenetic inheritance causes phenotypic changes in its offspring (Pigliucci, 2001). If the phenotypic trait an individual expresses is assumed to consist of a breeding value, determined by its genotype potentially at very many loci, and an environmental component (Falconer, 1960), phenotypic plasticity and nongenetic inheritance occur when environmental change alters the value of the environmental component of the phenotype (Via & Lande, 1985). Such dynamics are captured by reaction norms that describe how environmental variation influences phenotypic trait expression within a genotype (Falconer, 1990; Lande, 2009). If phenotypic plasticity or nongenetic inheritance change the distribution of phenotypic traits, this can alter survival and recruitment rates – and consequently population dynamics and selection. Such dynamics can occur even if the fitness functions themselves are not altered by environmental variation (Coulson et al., 2017). Phenotypic plasticity and genetic inheritance consequently have the potential to generate eco‐evolutionary feedbacks in the presence of unchanging fitness functions, with human‐induced environmental change having considerable potential to be a major driver of such feedbacks. The question we ask here is how does the impact of human‐induced environmental change on the environmental component of the phenotype influence eco‐evolutionary dynamics, and the way populations respond to environmental change? Our results extend to any type of environmental change, but we couch this paper in terms of human‐induced change, and in particular in the impacts of a deteriorating environment.

Environmental variation can have substantial effects on phenotypic trait values as is widely appreciated in statistical quantitative genetics – a powerful framework for studying evolution (Falconer, 1960; Lynch & Walsh, 1998). In quantitative genetic analyses, it is often essential to fit variables into statistical models to correct for environmental influences on phenotypic trait expression (Kruuk, 2004; Merilä et al., 2001). For example, variables such as population density or weather attributes – that often show temporal trends as a result of human activity – are sometimes fitted as fixed effects into animal models of free‐living populations (Fletcher et al., 2015; Kruuk et al., 2002; Potter et al., 2021), or year is fitted as a random effect (Kruuk, 2004; Lynch & Walsh, 1998). These environmental variables statistically adjust for reaction norms, allowing more robust estimates of additive genetic (co)variances by comparing phenotypic trait values amongst individuals of known relatedness once the effect of environmental variation on phenotypic trait values has been accounted for.

In predictive models widely applied to empirical systems, such as the breeder's equation, the additive genetic (co)variances are used to make evolutionary predictions but the evolutionary effects of environmental variation on phenotypic trait distributions are usually not incorporated (Chevin et al., 2010). We know that models such as the breeder's equation can provide accurate estimates of evolution over a single generation, but given the potential effects of environmental variation on selection via phenotypic plasticity and nongenetic inheritance, and via impacts on the fitness functions directly, these approaches may fail for the longer‐term predictions required to understand how anthropogenic environmental change will impact populations (Morrissey et al., 2010). This leads us to pose the following hypothesis: to make multi‐generational predictions of evolutionary change for populations in human‐induced deteriorating environments it is necessary to model the effects of the environment on the dynamics of both the breeding value (via selection) and environmental component of the phenotype (via selection, phenotypic plasticity and nongenetic inheritance). We test this prediction by constructing simple evolutionarily explicit Integral Projection Models (hereafter called EE‐IPMs) (Childs et al., 2016; Coulson et al., 2017; Rees & Ellner, 2019).

Integral projection models (IPMs) are discrete‐time population models structured by one or more continuous traits (Ellner et al., 2016). In addition, they can be structured by discrete characteristics such as age or sex (Ellner & Rees, 2006; Schindler et al., 2015). The models are constructed from mathematical functions typically identified from statistical analyses. These functions describe (i) associations between the values of one or several phenotypic traits measured at time *t* and per‐time step fitness (the fitness function) and (ii) phenotypic transitions between time *t* and *t* + 1 (transition functions) (Ellner et al., 2016). Most applications of IPMs assume time steps that are shorter than the generation length of the species being modelled. The fitness functions are typically divided into (i) the expected survival from *t* to *t* + 1 (the survival function), and (ii) the expected number of offspring produced between *t* and *t* + 1 that survive to recruit to the population at *t* + 1 (the recruitment function), while the transition functions are split into (iii) the values of trait(s) measured between *t* and *t* + 1 amongst surviving individuals (the development function) and (iv) the values of trait(s) measured in offspring when they recruit to the population at time *t* + 1. This last function has been referred to as the inheritance function by some authors (Coulson et al., 2010), and this has caused confusion (Chevin, 2015). We refer to it here as the parent–offspring phenotypic similarity function. IPMs can also be constructed on a per‐generation time step, where the recruitment function describes the association between a phenotypic trait and lifetime reproductive success, and the parent–offspring phenotypic difference function describes phenotypic trait similarity between parents and their offspring (Coulson et al., 2018). We use per‐generation time step models here. Regardless of the approach, functions can be statistically estimated from individual‐based phenotypic trait and demographic data that are used in statistical quantitative genetics and can include fixed and random effects describing how elements of the biotic or abiotic environment affect associations between trait values and each response variable (Coulson, 2012; Ellner et al., 2016).

The statistical functions are then combined to produce a projection model that iterates forward the distribution of phenotypic trait values from time *t* to time *t* + 1 (Easterling et al., 2000). At each time *t*, the projection model is usually approximated as a Lefkovich stage‐structured matrix. The random and fixed effects identified in the statistical analyses of each function can be included in the projection model if the modeller desires so or else can be ignored (Coulson, 2012; Ellner et al., 2016). If the model includes elements of the biotic and abiotic environment, including human‐induced environmental trends, the values in each matrix at each time *t* may vary between successive time steps. This generates a series of time‐varying matrices that can often be analysed using approaches from random matrix theory (Tuljapurkar, 2013; Tuljapurkar & Caswell, 2012).

In evolutionarily explicit IPMs the phenotypic trait distribution is described as a multivariate distribution of components of the phenotypic trait(s) involved – for example each trait is decomposed into a bivariate distribution of the breeding values and the environmental components (Childs et al., 2016; Coulson et al., 2017; Rees & Ellner, 2019). Selection operates on the phenotypic trait(s) under study, and this selection is then transmitted to each component of the phenotype. In models where the time step is shorter than the generation length, the breeding values remain fixed within individuals as they age, while, if desired, the environmental component of the phenotype may vary with the environment, generating phenotypic plasticity. The breeding values are genetically inherited (Childs et al., 2016), and assumptions about the effects of selection and inheritance on the additive genetic variance need to be explicitly specified (Coulson et al., 2017). If desired, the environmental component of the phenotypic trait(s) in offspring can be either random, a function of that of their parents (nongenetic inheritance), or dependent upon the abiotic or biotic environment experienced by the offspring. So far, the first option has been most commonly used (Childs et al., 2016; Coulson et al., 2017; Simmonds et al., 2020). Routinely, random developmental noise is incorporated into functions describing the dynamics of the environmental component of the phenotype. EE‐IPMs consequently provide a modelling framework where the environmental factors that were part of statistical quantitative genetic analyses can be included in models if desired, yet models can be constructed that are consistent with the breeder's equation where such environmental variation is not explicitly incorporated into predictions (Simmonds et al., 2019). EE‐IPMs thus allow researchers to examine how environmental variation, such as human‐induced deteriorating environmental trends, can impact evolution over multiple generations, including in anthropogenically modified environments.

Evolutionarily explicit Integral Projection Models are quite complex to construct and analyse (Childs et al., 2016), and there is a gap in the literature describing how simple versions of these models can be quite easily constructed. We attempt to fill this gap here. In doing this, we provide some novel biological insight by demonstrating how evolution will be fastest when it is cryptic, and slowest when phenotypic plasticity and nongenetic inheritance are adaptive.

Statistical quantitative genetics and structured population modelling both use similar data, and both have achieved considerable success in shining light on the complex patterns of phenotypic trait evolution, life‐history evolution and demographic changes observed in the wild. EE‐IPMs have been parameterized with estimates obtained through application of the animal model (Childs et al., 2016; Simmonds et al., 2020). Despite this, the two approaches have largely independent histories of development, different lexicons, cite different literatures, and are used by different communities. As a consequence, crosstalk between advocates of the two approaches is not as frequent or constructive as it could be. We do not claim that our approach is the only way to link structured population modelling and quantitative genetics nor that our integration is complete. We also do not generate new theory. Our aims, instead, are (i) to illustrate connections between the two approaches and thereby to, hopefully, encourage crosstalk, and (ii) explore how a human‐induced deteriorating environments might be expected to impact evolutionary trajectories.

## 2 METHODS

### 2.1 Modelling approach

**FIGURE 1:**
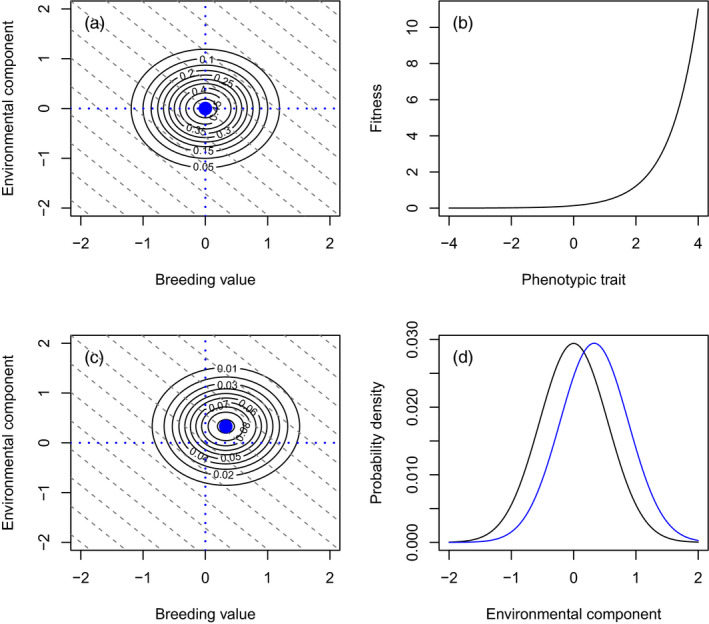
Action of selection on a phenotypic trait described as a bivariate distribution of *A* and *E*. (a) A bivariate distribution with $\bar{A}=\bar{E}=0$ and *σ_AA_* = *σ_EE_* = 0.5. Each dotted diagonal grey line represents the same value of the phenotype *z* = *A* + *E*. The dotted blue lines represent *y* = 0 and *x* = 0 and repeated in (c). (b) A fitness function, where life reproductive success increases exponentially with the value of the phenotype. (c) The outcome of applying the fitness function in (b) to the distribution in (a). The mean $\bar{A}$ and $\bar{E}$ have both changed, but the *σ_AA_* and *σ_EE_* have not. (d) The conditional distribution of *E* prior to selection in (a) and postselection in (c)

In general, EE‐IPMs assume that (Childs et al., 2016; Coulson et al., 2017; Rees & Ellner, 2019):

1. An individual *i's* phenotypic trait value *z_i_* is the sum of a breeding value *A_i_* and an environmental component *E_i_*. The bivariate distribution of the components of a hypothetical phenotypic trait is given in Figure 1a.
2. The environmental component of the phenotype can be determined by random developmental noise and aspects of the external abiotic or biotic environment **θ**. Note that such effects are frequently corrected for in quantitative genetic statistical analyses, but are rarely incorporated into predictive models (Chevin et al., 2010). A temporally deteriorating environment, caused, for example, by the establishment of an invasive species, or global warming, could consequently result in a negative trend across generations in the mean of the environmental component of the phenotype. In our modelling approach, we are agnostic to the developmental mechanisms underpinning a negative trend in the mean of the environmental component of the phenotype attributable to a deterioration in the environment.
3. Selection operates on phenotypic traits (Figure 1b for a hypothetical example), altering (i) the distribution of the phenotypic trait, (ii) the bivariate distribution of the *A* and *E* components of the trait (Figure 1c) and (iii) the conditional distributions of *A* and *E* prior to and postselection (Figure 1d for the change in the conditional distribution of *E* that is attributable to selection).
4. Aspects of the biotic or abiotic environment **θ** can also influence fitness. For example, individuals with the same phenotypic trait value may produce different numbers of offspring in good and bad environments.
5. Breeding values are genetically inherited such that the mean of the parental mid‐point breeding value distribution is the same as the mean of the breeding value distribution of the next generation of offspring. We will consider specific assumptions about the dynamics of the additive genetic variance in further detail below.
6. Nongenetic inheritance can occur when there is an association between the environmental components of parental phenotypes and the environmental components of offspring phenotypes. Note that such effects are sometimes corrected for in quantitative genetic statistical analyses as parental environmental effects (Lynch & Walsh, 1998) but are rarely incorporated into predictive models. In the models we construct here, we include nongenetic inheritance yet are silent on its underlying mechanistic causes.

We also make more assumptions specific to the models we report in this paper:

- Individuals have an annual life history and survive for one time step only. We consequently only construct models on a per‐generation time step.
- Phenotypic data are collected at birth, and the reproductive success of individuals alive at time *t* is estimated from matching newborn young at time *t* + 1 to their parents at time *t*. Fitness is consequently lifetime reproductive success.
- The environment **θ** can vary with time, influencing the mean value of the environmental component of the phenotype in each generation.

The bivariate distribution of *A* and *E* at time *t*, *N* (*A*, *E*, *t*), is operated on by a kernel, $\phi \left(\left(A^{\prime },E^{\prime }\right)|\left(A,E\right),t\right)$, that describes all possible transition rates from (*A*, *E*) at time *t* to (*A*′, *E*′) at time *t* + 1, including those that occur at rate zero because they are biologically impossible. The primes here depict that the values of *A* and *E* can change between parents and their offspring. The model can be written as follows:

$$N\left(A^{\prime },E^{\prime },t+1\right)=\iint \phi \left(\left(A^{\prime },E^{\text{′}}\right)|\left(A,E\right),t\right)N\left(A,E,t\right)dAdE.$$

The integral limits are taken to be below and above all possible values of *A* and *E* but are not displayed to simplify notation (Ellner et al., 2016). In addition, from now on we simply use a single, rather than a double, integral sign, with the reader determining the variables over which the integral is taken by the infinitesimals on the far‐right hand side (*dA* and *dE*).

The kernel $\phi \left(\left(A^{\prime },E^{\prime }\right)|\left(A,E\right),t\right)$ is constructed to include the biological processes that determine the dynamics of the bivariate distribution of *A* and *E* and their drivers. The ‘trick’ in formulating a model is to specify the functions and the rules they encode. In our simple model, we will consider a fitness function $R\left(A+E,\theta ,t\right)$, that determines the strength and direction of selection, followed by a parent–offspring phenotypic difference function $D((A^{\prime },E^{\prime })|(A,E),\theta ,t)$ (the transition function) that captures the genetic inheritance of breeding values and various nongenetic inheritance processes that can influence the dynamics of the environmental component of the phenotype. Consequently, $\phi \left(\left(A^{\prime },E^{\prime })|\left(A,E\right),t\right)\right)=D\left((A^{\prime },E^{\prime })|(A,E),\theta ,t\right)R\left(A+E,\theta ,t\right)$ and

(1) $$N\left(A^{\prime },E^{\prime },t+1\right)=\int \left[D\left((A^{\prime },E^{\prime })|(A,E),\theta ,t\right)R\left(A+E,\theta ,t\right)\right]N\left(A,E,t\right)dAdE,$$

noting the implications of the notation change described above.

The functions $D((A^{\prime },E^{\prime })|(A,E),\theta ,t)$ and $R\left(A+E,\theta ,t\right)$ do not commute, which means their order matters. We consequently treat selection operating first, following by the transmission of breeding values and environmental components of the phenotype between parental and offspring values. This means that $D((A^{\prime },E^{\prime })|(A,E),\theta ,t)$ is conditional on reproduction: if you do not reproduce you will not contribute to the components of the phenotypic traits in offspring.

The fitness function $R\left(A+E,\theta ,t\right)$ informs that lifetime reproductive success is determined by the phenotypic trait value *z* = *A* + *E*, and the potentially multidimensional environment, **θ**, experienced at time *t*. The form of this function will combine with the phenotypic variance to determine the strength of selection.

The fitness function can be thought of as operating on the distribution of *N* (*A*, *E*, *t*) to produce a bivariate distribution of *A* and *E* postselection: $N_{s}\left(A,E,t\right)=R\left(A+E,\theta ,t\right)N\left(A,E,t\right)$. The means of both the breeding value and the environmental component of the phenotype in this bivariate distribution will differ from the means of these quantities in *N* (*A*, *E*, *t*) if (i) selection is directional (i.e., the slope of the phenotype on fitness in the fitness function *R* (*A* + *E*, **θ**, *t*) is nonzero) and (ii) the additive genetic variance and the variance in the environmental component of the phenotype are both greater than zero.

**FIGURE 2:**
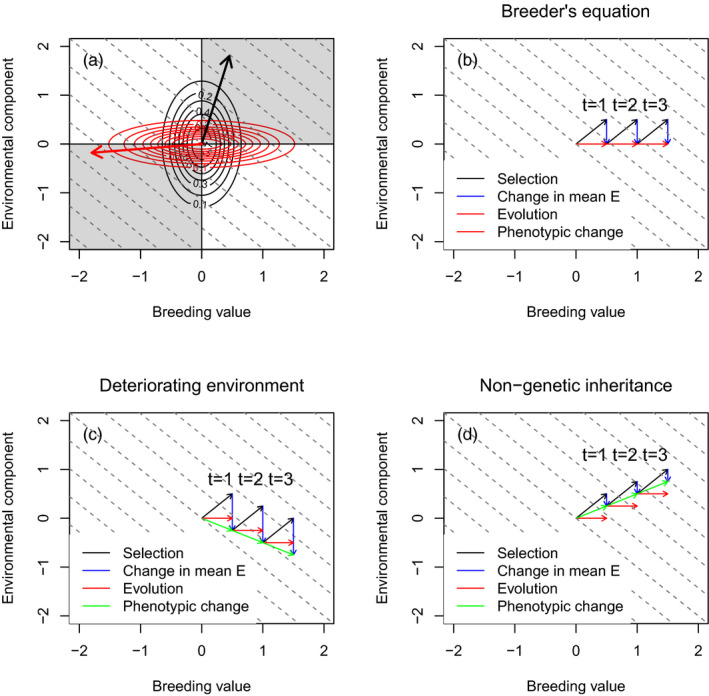
Selection and evolution. (a) When selection operates on the phenotype *z*, selection on *A* and *E* is always in the same direction. The black lines represent a bivariate distribution of *A* and *E* where $\sigma_{\mathrm{AA}}<\sigma_{\mathrm{EE}}$. The black vector (arrow) shows the direction of evolution if selection is directional and positive. The length of the arrow is arbitrary in this hypothetical example but in real numerical examples would represent the strength of selection. The red lines represent a bivariate distribution of *A* and *E* where $\sigma_{\mathrm{AA}}>\sigma_{\mathrm{EE}}$. The red vector (arrow) shows the direction of evolution if selection is directional and negative. (b) The dynamics of the breeder's equation, where selection shifts the phenotype by altering both $\bar{A}\left(t\right)$ and $\bar{E}\left(t\right)$ black arrow. Rules of genetic inheritance are such that $\bar{A_{s}}\left(t\right)=\bar{A}\left(t+1\right)$ (red arrow). Rules of nongenetic inheritance mean that all gains made by selection on $\bar{E_{s}}\left(t\right)$ are lost and $\bar{E}\left(t+1\right)=\bar{E}\left(t\right)=0$ (blue arrow). In this case, change in the mean phenotype equals change in the mean breeding value $\Delta \bar{Z}\left(t\right)=\Delta \bar{A}\left(t\right)$. (c) When the environment deteriorates, and this leads to a temporal trend in the environmental component of the phenotype, evolutionary change (red lines) can be countered by change in $\bar{E}$ (blue lines) that more than reverses any gains made by selection (black lines). The phenotypic trait mean (green line) can change in a direction opposite to that of selection. (d) When nongenetic inheritance is adaptive, it moves the mean phenotype (green line) in the same direction as selection. In this example, only a fraction of the effect of selection on the environmental component of the phenotype is passed across generations by nongenetic (compare black and green lines)

Because we assume that selection operates on the phenotypic trait and that the components of the phenotype add together to determine its value within an individual (*z_i_* = *A_i_* + *E_i_*), this means that directional selection must displace $\bar{A}$ and $\bar{E}$ in the same direction, even if their rates of change differ (Figure 2a). We consider two hypothetical cases in Figure 2a – in black, directional selection is positive, and the additive genetic variance is less than the variance of the environmental component of the phenotype. In contrast, in red, directional selection is negative, and the additive genetic variance is greater than the variance of the environmental component of the phenotype. Positive directional selection can only shift the $\bar{A}$ and $\bar{E}$ into the upper right quadrant in Figure 2a while negative directional selection can only shift $\bar{A}$ and $\bar{E}$ into the lower left quadrant. Selection alone cannot move $\bar{A}$ and $\bar{E}$ in contrasting directions. Note that within the shaded quadrants that selection can explore, the angle of the vectors is determined by the ratio of the additive genetic variance to the variance of the environmental component of the phenotype.

We next turn to the second function in the kernel $\phi \left(\left(A^{\prime },E^{\prime }\right)|\left(A,E\right),t\right)$, which describes the ‘map’ between parental values of *A* and *E* and offspring values of *A*′ and *E*′. We write this function $D(A^{\prime },E^{\prime }|A,E,\theta ,t)$. The symbol ‘|’ means ‘given’. So, *A*′ and *E*′ take their values given *A*, *E* and **θ** at time *t*. This function is a probability density function, such that all possible transitions out of location (*A*, *E*) sum to unity (Easterling et al., 2000).

We now need to specify this parent–offspring phenotypic difference function $D(A^{\prime },E^{\prime }|A,E,\theta ,t)$ to capture specific rules. The first biological rule we need to respect is genetic inheritance for breeding values. This means that the mean of the breeding values in offspring, $\bar{A^{\prime }}$, must be equal to the mean of mid‐point breeding values of each offspring's parental pair, $\bar{A_{s}}$, where the subscript *s* means postselection (Falconer, 1990). Next, because we want our model to be dynamic and to make predictions over multiple generations, we need to make assumptions about the dynamics of the variance of breeding values (the additive genetic variance), and how that changes (or not) from one generation to the next (Lande, 2009; Turelli & Barton, 1994). There are four ways in which the dynamics of the variance have been treated in structured models (Table 1), and the choice will depend upon the assumptions the researcher wishes to make. Arguably the most intuitive way to generate the offspring distribution from the quantitative genetic perspective is to work with an algorithm as follows (approach 1 in Table 1):

1. Take the conditional distribution of selected parental breeding values (each parental *A* is represented by the number of offspring it produces) (Childs et al., 2016)
2. Assume random mating and an identical demography for males and females (easily relaxed but the maths becomes more involved). These assumptions mean we only need to track the dynamics of a single distribution of *A* and *E* containing both males and females.
3. Convolve the distribution of selected parents from step 1 with itself to generate a distribution of mid‐point values of *A*.
4. Convolve the distribution in 3 with a Gaussian distribution with a mean of 0 and a variance that captures the segregation variance to produce a distribution of offspring *A*′.

**TABLE 1:** Four approaches have been used in evolutionarily structured models to determine the dynamics of the additive genetic variance. Approach 1 and 2 give indistinguishable results in cases where they have been compared, and these are similar to those obtained in approach 3 (Coulson et al., 2017)

Approach	Comment	Reference
1. Convolve distributions of parental breeding values to generate a distribution of mid‐point breeding values. Convolve this distribution with a Gaussian distribution with a mean of zero to add segregation variance.	Additive genetic variance in each generation can be non‐Gaussian	Childs et al. (2016) and Coulson et al. (2011)
2. Construct a linear and Gaussian probability density function (typical of a standard IPM) that passes through the point $\left(\bar{A_{s}},\bar{A_{s}}\right)$, has a slope of 0.5, and generates a constant variance.	Additive genetic variance in each generation can be non‐Gaussian	Coulson et al. (2017)
3. Generate a Gaussian distribution of breeding values in offspring with a constant variance and a mean equal to the mean of the breeding value distribution postselection ($\bar{A_{s}}$).	Additive genetic variance in each generation is Gaussian	Lande (1982) and Simmonds et al. (2020)
4. Allow selection to erode the additive genetic variance.	Additive genetic variance in each generation can be non‐Gaussian	Coulson et al. (2017)

An alternative approach is to simply assume that the distribution of breeding values amongst offspring is always Gaussian and has a distribution with a mean breeding value $\bar{A_{s}}$, and a constant variance that does not change with time (Lande, 1982, 2009). The two approaches differ and produce slightly different dynamics, because the approach based on convolutions does not necessarily produce a Gaussian distribution of offspring breeding values.

Next, we turn to rules for the environmental component of the phenotype. There may be three aspects we wish to incorporate into the postselection dynamics of *E*. First, random developmental noise (Figure 2b) with a mean of zero and a fixed variance; second, the effects of the abiotic or biotic value in year *t* on the mean of the environmental component of the phenotype (Figure 2c) – that is, the processes that generate reaction norms (Chevin et al., 2010; Reed et al., 2011); and third, nongenetic inheritance – that is, a correlation between parental and offspring environmental components of the phenotype caused by nongenetic inheritance (Figure 2d) (Salinas et al., 2013). If we can incorporate these processes into our model, we can examine the effects the environmental effects often included in statistical quantitative genetic analyses on evolutionary dynamics. In this paper, to keep things simple, we focus on the first two processes only.

Let us start with the assumption that the environmental component of the phenotype is determined solely by random developmental noise. The distribution of the environmental component of the phenotype in offspring will be independent of the distribution of the environmental component of the phenotype in parents, will have a mean of 0, and a constant variance across generations. This would generate the temporal dynamics of *A* and *E* shown in Figure 2b.

Next, we turn to the case where there is a trend in some component of the biotic or abiotic environment **θ** (Kruuk et al., 2002). Such a trend could be caused by human‐induced environmental, such as the establishment of an invasive species or global warming. In each generation let us assume that the value of **θ** gets smaller, and this results in a decrease in the mean of the environmental component of the phenotype because *E* is a function of **θ** – that is, we are describing a reaction norm where the average phenotypic trait value across all breeding values changes as the environment changes (i.e., this is not a genotype‐by‐environment interaction, but rather the phenotypes expressed by each genotype or breeding value are impacted in identical ways). In other words, we are incorporating the effect of a trending fixed effect that is found to influence the mean of a phenotypic trait value in a statistical quantitative genetic analysis. We continue to assume positive selection. In this case, we would generate the type of phenotypic trait dynamics displayed in Figure 2c.

Finally, we turn to the case where there are maternal effects, or nongenetic inheritance, such that there is a correlation between environmental components of the parental and offspring phenotypes (Lynch & Walsh, 1998). The similarity between parental and offspring environmental components of the phenotype can generate the types of dynamics depicted in Figure 2d.

We will give examples of the form and parameterization of these functions below. But before we do, we describe the steps required to implement an EE‐IPM.

### 2.2 Model implementation

We approximate Equation 1 into matrix form: $N\left(t+1\right)=D\left(t\right)R\left(t\right)N\left(t\right)$ (Easterling et al., 2000). First, we need to approximate the bivariate distribution *N* (*A*, *E*, *t*) by categorizing it into many small bins to generate the column vector **N** (*t*). In Table 2, below we use 10,000 bins. Each value in **N** (*t*) describes the number of individuals in each (*A*, *E*) discrete category at time *t*. The mid‐point values of each (*A*, *E*) category is described in columns 3 and 4 of Table 2. We consider values of *A* and *E* ranging from 1 to 10 with 100 categories for both. Note that these values could be centred on zero if desired, as depicted in Figures 1 and 2. Centering is useful in statistical analyses, but it does not influence the construction or iteration of EE‐IPMs. We show the values of the phenotypic trait *z* which are determined by summing the values of *A* and *E*.

**TABLE 2:** Describing a bivariate distribution of and as a column vector

Element number	**N** (*t*)	Value of *A*	Value of *E*	Value of *z* = *A* + *E*
$\begin{array}{c} 1\\ 2\\ 3\\ \ldots \\ 100\\ 101\\ 102\\ \ldots \\ 10,000 \end{array}$	$\left[\begin{array}{c} 0\\ 0.015\\ 0.024\\ \ldots \\ 0.021\\ 0.037\\ 0.012\\ \ldots \\ 0 \end{array}\right]$	$\begin{array}{c} 1\\ 1\\ 1\\ \ldots \\ 1\\ 1.1\\ 1.1\\ \ldots \\ 10 \end{array}$	$\begin{array}{c} 1\\ 1.1\\ 1.2\\ \ldots \\ 10\\ 1\\ 1.1\\ \ldots \\ 10 \end{array}$	$\begin{array}{c} 2\\ 2.1\\ 2.2\\ \ldots \\ 11\\ 2.1\\ 1.2\\ \ldots \\ 100 \end{array}$

We specify the vector **N** (*t* = 1) as bivariate normal with two means (one each for $\bar{A}$ and $\bar{E}$) and a variance–covariance matrix $\Sigma =\left[\begin{array}{cc} \sigma_{\mathrm{AA}} & \sigma_{\mathrm{EA}}\\ \sigma_{\mathrm{AE}} & \sigma_{\mathrm{EE}} \end{array}\right]$ that can be estimated from statistical analyses used in quantitative genetics (Falconer, 1960; Lynch & Walsh, 1998). The fitness function *R* (*A* + *E*, **θ**, *t*) can be identified by the statistical analysis of phenotypic trait and reproductive success data. For example, if it is linear, it would take the form

$$R\left(A+E,\theta ,t\right)=\beta_{0}+\beta_{z}z+\ldots +\beta_{\theta_{i}}\theta_{i},$$

where the *β_x_* s are statistically estimated parameters, and ***θ**_i_* is a variable used to characterize one aspect of the environment **θ**. The function does not need to be linear (e.g., Figure 1b is exponential) but can be of any parametric or nonparametric form the researcher chooses. Fitness predictions from $R\left(A+E,\theta ,t\right)$ for each of the categories used to construct **N** (*t*) (column 5 of Table 2) are then used to construct a diagonal matrix **R** (*t*) describing the fitness of each *z* = *A* + *E*. The matrix **R** (*t*) is square and with the same length and width dimensions as the length of **N** (*t*) (e.g., 10,000 in our example).^1^ Note, also, that the value of **R** (*t*) for a value of *A* = 2 and *E* = 3 would be the same as the value of **R** (*t*) for *A* = 2.5 and *E* = 2.5 as both give the same phenotypic trait value of *z* = *A* + *E* = 5.

The kernel $D(A^{\prime },E^{\prime }|A,E,\theta ,t)$ can also be approximated as a matrix **D** (*t*) with the same dimensions as **R** (*t*). This matrix describes the probability of transition from all parental phenotypic trait component values (*A*, *E*) to all offspring phenotypic trait components values (*A*′, *E*′). In our example, the top left element of the matrix would describe the transition probability from (*A* = 1, *E* = 1) to (*A*′ = 1, *E*′ = 1); the cell in the top row and second column describes the transition probability (*A* = 1, *E* = 1.1) to (*A*′ = 1, *E*′ = 1); the first cell in the second row describes the transition probability (*A* = 1, *E* = 1) to (*A*′ = 1, *E*′ = 1.1) etc.

It is not always computationally necessary to construct the matrix **D** (*t*) and it can be significantly computationally faster to construct the vector **N** (*t* + 1) directly from **N_s_** (*t*) = **R** (*t*) **N** (*t*). We illustrate this here, because for readers who are unfamiliar with Lefkovitch matrices their construction can be opaque, and we do not have space to elaborate here.

The following algorithm could be used to construct the vector **N** (*t* + 1), removing the need to populate the matrix **D** (*t*):

1. Calculate the sum $n\left(t+1\right)=\sum N_{s}\left(t\right)$ where *n* (*t* + 1) is the population size at birth of the next generation.
2. Calculate the mean of $\bar{A_{s}}=\sum {\mathrm{AN}}_{s}\left(t\right)/\sum N_{s}\left(t\right)$ where **A** is a column vector of the values of *A* in the third column of Table 1.
3. Choose your assumption about the effect of selection on the additive genetic variance. We will assume that it remains Gaussian with a constant variance *σ_AA_*.
4. Given (3), generate a Gaussian probability distribution with a mean of $\bar{A_{s}}$ and a variance *σ_AA_* discretized into the number of unique bins used to categorize the distribution of *A*. Call this $N_{A}^{\mathrm{short}}\left(t+1\right)$.
5. Replicate each value within $N_{A}^{\mathrm{short}}\left(t+1\right)$ by the number of unique bins used to categorize *E* (4th column, Table 1) to generate $N_{A}^{\mathrm{long}}\left(t+1\right)$. This will generate the ‘blocks’ of breeding values with the same value depicted in Table 2 (third column?).
6. Standardize the vector produced in (5) to sum to unity.
7. Within each block of values of *A* in $N_{A}^{\mathrm{long}}\left(t+1\right)$ (Table 2), we now need to distribute densities of each of the values of *E*. Generate a discrete Gaussian probability density distribution with a mean $\bar{E}$ (that may be determined by the value of **θ** or parental values of *E*, and a variance equal to *σ_EE_*). Call this vector $N_{E}^{\mathrm{short}}\left(t+1\right)$.
8. Replicate the vector **N_E_** (*t* + 1) by the number of unique bins used to categorize *E* to generate $N_{E}^{\mathrm{long}}\left(t+1\right)$.
9. Standardize $N_{E}^{\mathrm{long}}\left(t+1\right)$ to sum to unity.
10. Multiply the two vectors produced in (5) and (8) and standardize to sum to *n* (*t* + 1). This produces **N** (*t* + 1).

The code to implement these algorithms is provided on Zenodo.

### 2.3 Choice of parameters

In this section, we describe the two models we use in this paper. The parameters we choose are simply for illustration, and the general results we report are not specific to the parameterization although the specific rates are. We do not identify parameter values from statistical analyses. Simmonds et al. (2019), Simmonds et al. (2020) show how parameters from the analysis of data can be used to parameterize EE‐IPMs.

We use 500 bins for both *A* and for *E* and upper and lower integration limits for each of 0 and 40. We define the initial bivariate distribution of *N* (*A*, *E*, *t* = 1) as Gaussian with $\bar{A}=\bar{E}=18$ and $\Sigma =\left[\begin{array}{cc} 2 & 0\\ 0 & 2 \end{array}\right]$. In both models, we specify a constant fitness function *R* (*A* + *E*, *t*) = −2.5 + 0.1*z* that is constant with time and not impacted by environmental variation. Although this function would generate negative values of fitness for *z* < 25, we have chosen parameter values to ensure that this does not happen. Alternatively, we could choose a nonlinear function that remains within bounds.

In model 1, we assume that $\bar{E}=18$ and *σ_EE_* = 2. This is equivalent to saying that the environmental component of the phenotype is determined solely by developmental noise and remains constant in the phenotypic trait distribution of offspring from one generation to the next. The choice of a constant value of $\bar{E}$ is irrelevant for the dynamics. Model 1 is a dynamic version of the univariate breeder's equation.

In model 2, we assume a reaction norm where the phenotypic trait value produced by all breeding values is a function of a temporally deteriorating environment. We define the mean environmental component as $\bar{E}=18‐0.5t$ with *σ_EE_* = 2 in each new generation. This would capture the statistical effect of a human‐induced trending environment that influences the mean phenotypic trait value in a statistical analysis of phenotypic similarity between relatives.

## 3 RESULTS

**FIGURE 3:**
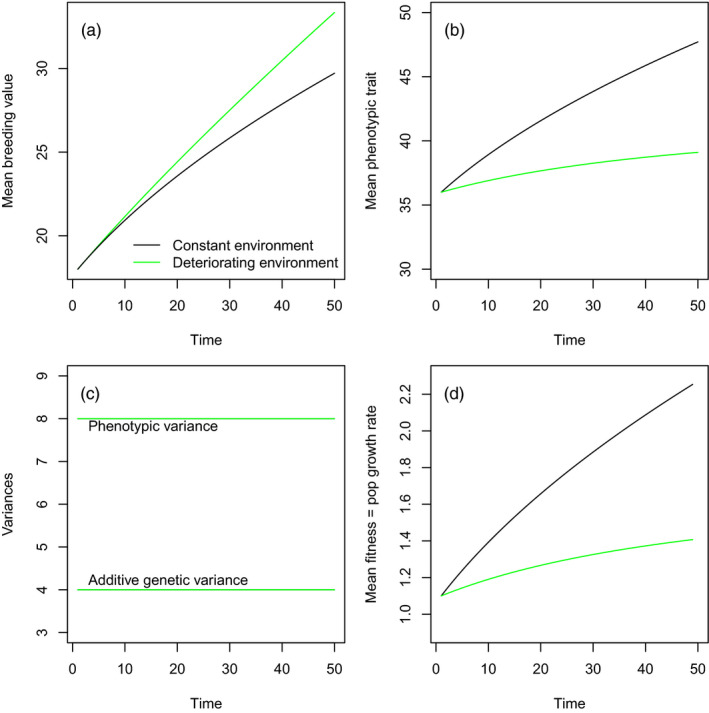
Dynamics of a model where the environment remains constant (black lines) and deteriorates with time (green lines) impacted the mean of the environmental component of the phenotype. (a) Evolution occurs fastest in the deteriorating environment than in the constant environment, (b) the mean value of the phenotypic trait changes fastest in the constant environment, (c) the phenotypic variance and additive genetic variance (and consequently the variance in the environmental component of the phenotype) remain constant with time (the green lines obscure black lines in this plot) and (d) the dynamics of mean fitness

When the mean environment deteriorates with time – perhaps due to human‐induced environmental change – and this trend influences the value of $\bar{E}$ (model 2), evolution (defined as change in $\bar{A}$) occurs at a faster rate than when $\bar{E}$ remains constant with time (model 1; Figure 3a). In our example, evolution is consequently faster in a human‐induced deteriorating environment than in a constant one. The reason for this is that the mean phenotype $\bar{z}=\bar{A}+\bar{E}$ changes more slowly when $\bar{E}$ trends downwards than when it does not (Figure 3b). In both our models, the additive genetic variance, the variance in the environmental component of the phenotype, and the phenotypic variance remain constant with time (Figure 3c). However, the population growth rate (which equals mean lifetime reproductive success) evolves much more slowly when the environment deteriorates over time compared to when it does not. It is the contrasting dynamics of the population growth rate that generates the difference in rates of evolution between the two models, with the difference in the population dynamics driven by the temporal dynamics of $\bar{E}$. All other aspects of the two models are identical.

## 4 DISCUSSION

Our aim here is to make evolutionarily explicit IPMs accessible to readers who do not have a background in structured population modelling and to explore how a deteriorating environment that mimics the effect of human‐induced biotic or abiotic change influences evolutionary dynamics. We have done this by (i) providing background that has not been explicitly described in previous papers using EE‐IPMs and (ii) introducing very simple models, one of which includes the effects of a deteriorating on the environmental component of the phenotype. Nonetheless, even these simplified models provide interesting insight.

In both our models, we have a constant, linear, fitness function. The only difference between our two models is that one contains a deteriorating environment designed to mimic human‐induced environmental change that impacts the mean of the environmental component of the phenotype, while the other does not. Such effects of the mean environment on the mean value of phenotypic traits are well‐documented in statistical analyses used by statistical quantitative geneticists (Kruuk, 2004; Kruuk et al., 2002; Wilson et al., 2006) but are rarely incorporated into predictive models (Morrissey et al., 2010).

Why do these results arise? Model 1, where there is no environmental deterioration, generates dynamics like those depicted in Figure 2b: all phenotypic change can be attributed to selection and $\Delta \bar{z}=\Delta \bar{A}$. In contrast, in model 2, where there is a deteriorating environment, we observed dynamics like those depicted in Figure 2c. In this case, $\Delta \bar{z}\neq \Delta \bar{A}$ due to the trend in $\bar{E}$. However, in addition, the trend in $\bar{E}$ generates divergence in the dynamics of selection between the two models, and this leads to a difference in the rate of evolution.

**FIGURE 4:**
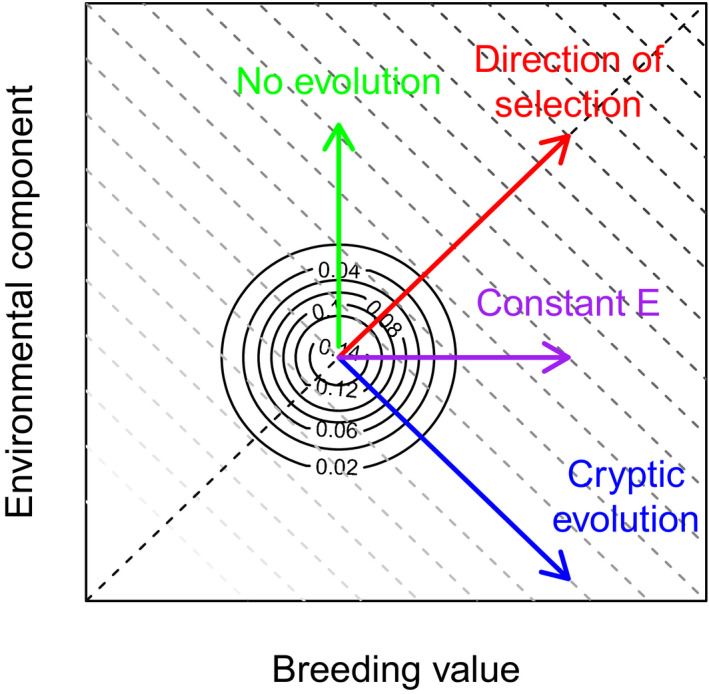
A hypothetic example of evolution in bivariate space helping summarize our results. The diagonal lines represent constant phenotypic trait value clines, with the darker colour representing larger trait values (and when fitness is directional and positive) higher fitness. Because the additive genetic variance equals the variance in the environmental component of the phenotype, the vector describing the direction of selection is at 45 degrees (red line). In both our models, selection is in this direction but the strength varies over time. The types of evolutionary dynamics model 1 produces are depicted by the purple arrow. In our second model, evolution is partly cryptic. When change in the dynamics of the mean breeding value is completely offset by nonadaptive change in the environmental component of the phenotype evolution is fast (not depicted), and the phenotypic trait does not change (remains on the same diagonal line) but its components change in opposing directions. The green line would represent a case where there is no additive genetic variance and all phenotypic change is attributable to the dynamics of the environmental component

A selection differential on a phenotypic trait can be written as $\frac{\text{cov}\left(z,w\right)}{\bar{w}}$ where *w*, in our model, is absolute lifetime reproductive success and $\bar{w}$ is mean lifetime reproductive success. For an annual life history, $\bar{w}$ is the population growth rate (Fisher, 1930). In both our models cov (*z*, *w*) remains constant with time – it is determined by the slope of our linear fitness function. However, $\bar{w}$ changes with time at different rates between our two models. The reason for this is we have a constant fitness function: mean fitness consequently increases with the mean of the phenotype. When there is a trend in $\bar{E}$ with time, there is consequently a trend in the mean phenotype with time, and hence, mean fitness will change at a different rate compared to when there is no trend in $\bar{E}$. When the direction of selection is positive, a negative trend in $\bar{E}$ will accelerate evolution, while a positive trend will slow it via its effect on mean fitness. For example, when nongenetic inheritance is adaptive it will accelerate the rate of change in mean fitness, and consequently decrease the selection differential, thus slowing the rate of evolution. In contrast, when nongenetic inheritance is maladaptive, it will decrease the rate of change in mean fitness and consequently accelerate the rate of evolution (Figure 4, and see also Coulson et al., 2017).

The dynamics of selection are rarely decomposed into the dynamics of the covariance between phenotypic traits and absolute fitness and the dynamics of mean fitness. However, doing this does allow some useful insight. For example, because evolution simultaneously alters the mean value of phenotypic traits and mean fitness (Fisher, 1930), it should not be assumed that selection is constant with time when making evolutionary predictions with a constant fitness function. Our results show that the dynamics of E – typically ignored in traditional quantitative genetic approaches, but potentially important when investigating human‐induced evolution – can change the denominator of the selection differential, by modifying the rate of change of mean fitness. Selection differentials can vary with time due solely to evolution of the population growth rate (Pelletier & Coulson, 2012).

Our models are deliberately very simplistic. In real settings, fitness functions are likely to include environmental variation and density dependence (Ellner et al., 2016; Simmonds et al., 2019, 2020). We also include only one phenotypic trait, but selection operates simultaneously on multiple traits (Lande & Arnold, 1983). Finally, mean fitness (the population growth rate) will fluctuate with time within a generation in iteroparous species (Coulson et al., 2005). EE‐IPMs have been constructed for multivariate phenotypic traits, for iteroparous species, and in both variable and deteriorating environments (Childs et al., 2016; Coulson et al., 2017; Simmonds et al., 2020). A wide range of more realistic settings on evolutionary dynamics can consequently be examined. In addition, IPMs can be used to simultaneously study not only evolutionary, phenotypic trait and population dynamics, but also the dynamics of life histories and interacting species (Bassar et al., 2017; Childs et al., 2004; Coulson et al., 2011; Ellner et al., 2016; Rees et al., 2014). These models are consequently quite flexible and can also be used to study eco‐evolutionary feedbacks and may be particularly relevant for human‐induced deterioration in the environment. Moreover, they are easily parameterized from data routinely used to conduct statistical quantitative genetic analyses and explicit genotype‐by‐environment interactions (where the environmental component of the phenotype is impacted in different ways by environmental change within different genotypes) can be easily incorporated.

Despite the positives of IPMs, they are not a panacea. To date, no one has constructed EE‐IPMs for environment‐specific phenotypic traits. Traits that are only expressed at specific ages have been incorporated into models (Coulson et al., 2017), and similar logic could be used for environment‐specific traits (Wilson et al., 2006). Second, quantitative geneticists often treat fitness as a phenotypic trait and are interested in the additive genetics of fitness. The evolution of fitness that is not coupled directly via fitness function to a specific trait has not yet been incorporated into an IPM and doing so will not be entirely straightforward, but is theoretically feasible. But perhaps the biggest limitation of IPMs is they do become computationally cumbersome as the size of the multivariate distribution being modelled increases (Ellner et al., 2016). Once the number of dimensions exceeds 6–10, high‐performance computing may be required to iterate models unless some way of avoiding multiplying large matrices together can be found (as we demonstrate in our models).

There are, of course, many other structured populations models that have been developed to examine evolutionary dynamics (Barfield et al., 2011; Charlesworth, 1994; Chevin et al., 2010; Lande, 1982) and nonstructured models assuming normality of the additive genetic variance (e.g., approach 4 in Table 1). Some consider reaction norm approaches to examine the effects of environmental change on dynamics (Lande, 2009). These valuable approaches have rarely been parameterized for real systems from statistical quantitative genetic analyses, and they do not link to explicit environmental drivers such as climatic variation as EE‐IPMs have been (Simmonds et al., 2020). Instead, models have assumed that different breeding values express different phenotypes in contrasting environments, without the driver of the contrast necessarily being included in models (Lande, 2009). The major difference between our approach and these other theoretical models, is that we explicitly model the dynamics of the environmental component of the phenotype and how it is impacted by environmental variation. However, in doing this, our approach is more intuitive, as it is straightforward to decompose the effects of environmental change on population, phenotypic trait and evolutionary dynamics, and on the feedbacks between these processes while still being consistent with the evolutionary assumptions incorporated into other modelling frameworks (e.g., Barfield et al., 2011; Charlesworth, 1994; Chevin et al., 2010; Lande, 1982).

It is our belief that structured models and statistical quantitative genetics are both powerful tools to study evolution. There are ways these approaches can be combined, and once they are they offer potential to shed light on evolutionary dynamics. Investigating both the statistical quantitative genetic and structured modelling literature is time consuming given that both are large, specialized and use different vocabularies. Nonetheless, collaboration rather than distrust between researchers in each discipline could pay dividends.

## CONFLICT OF INTEREST

There are no conflicts of interest to declare.

## Data Availability

There are no data used in this paper. Code to run models will be made available on Zenodo following acceptance.

## Footnotes

^1^
It is computationally faster to work with **R** (*t*) as a column vector and using element‐wise multiplication but we don't dwell on this here.
